# Extrusion 3D (Bio)Printing of Alginate-Gelatin-Based Composite Scaffolds for Skeletal Muscle Tissue Engineering

**DOI:** 10.3390/ma15227945

**Published:** 2022-11-10

**Authors:** Surendrasingh Y. Sonaye, Elif G. Ertugral, Chandrasekhar R. Kothapalli, Prabaha Sikder

**Affiliations:** 1 Mechanical Engineering, Cleveland State University, Cleveland, OH 44115, USA; 2 Chemical and Biomedical Engineering, Cleveland State University, Cleveland, OH 44115, USA

**Keywords:** bioprinting, alginate–gelatin scaffolds, skeletal muscle tissue engineering, volumetric muscle loss, rheology

## Abstract

Volumetric muscle loss (VML), which involves the loss of a substantial portion of muscle tissue, is one of the most serious acute skeletal muscle injuries in the military and civilian communities. The injured area in VML may be so severely affected that the body loses its innate capacity to regenerate new functional muscles. State-of-the-art biofabrication methods such as bioprinting provide the ability to develop cell-laden scaffolds that could significantly expedite tissue regeneration. Bioprinted cell-laden scaffolds can mimic the extracellular matrix and provide a bioactive environment wherein cells can spread, proliferate, and differentiate, leading to new skeletal muscle tissue regeneration at the defect site. In this study, we engineered alginate–gelatin composite inks that could be used as bioinks. Then, we used the inks in an extrusion printing method to develop design-specific scaffolds for potential VML treatment. Alginate concentration was varied between 4–12% *w*/*v*, while the gelatin concentration was maintained at 6% *w*/*v*. Rheological analysis indicated that the alginate–gelatin inks containing 12% *w*/*v* alginate and 6% *w*/*v* gelatin were most suitable for developing high-resolution scaffolds with good structural fidelity. The printing pressure and speed appeared to influence the printing accuracy of the resulting scaffolds significantly. All the hydrogel inks exhibited shear thinning properties and acceptable viscosities, though 8–12% *w*/*v* alginate inks displayed properties ideal for printing and cell proliferation. Alginate content, crosslinking concentration, and duration played significant roles (*p* < 0.05) in influencing the scaffolds’ stiffness. Alginate scaffolds (12% *w*/*v*) crosslinked with 300, 400, or 500 mM calcium chloride (CaCl_2_) for 15 min yielded stiffness values in the range of 45–50 kPa, i.e., similar to skeletal muscle. The ionic strength of the crosslinking concentration and the alginate content significantly (*p* < 0.05) affected the swelling and degradation behavior of the scaffolds. Higher crosslinking concentration and alginate loading enhanced the swelling capacity and decreased the degradation kinetics of the printed scaffolds. Optimal CaCl_2_ crosslinking concentration (500 mM) and alginate content (12% *w*/*v*) led to high swelling (70%) and low degradation rates (28%) of the scaffolds. Overall, the results indicate that 12% *w*/*v* alginate and 6% *w*/*v* gelatin hydrogel inks are suitable as bioinks, and the printed scaffolds hold good potential for treating skeletal muscle defects such as VML.

## 1. Introduction

Skeletal muscle injuries are some of the most common traumas in the military, sports, and civilian communities. Some of the most common injuries include in situ necrosis or rhabdomyolysis, where the myofibers are partially necrotized, but the basal lamina, mysial sheaths, and adjacent blood vessels remain intact [1]. More intense muscle injuries caused by contusion, strain, or laceration involve complete rupture of the muscle fibers, including the basal lamina, mysial sheaths, and nearby capillaries [1]. Under such injury conditions, skeletal muscles have the innate ability to regenerate and heal the wound. Although therapeutic interventions such as physical therapy are mandatory to enhance muscle regeneration, they are mostly confined to non-surgical treatments. 

In contrast, when an injury involves the loss of a substantial portion of muscle tissue, i.e., approximately >20% of the local tissue, it becomes almost impossible for the body to regenerate the lost muscle tissue. Such an injury is commonly known as Volumetric Muscle Loss (VML) and can occur due to traumatic events such as explosions, major falls, road accidents, or planned surgical ablation like tumor removal [2]. VML is one of the most debilitating musculoskeletal injuries in the military and civilian communities [3]. Estimates from civilian trauma indicate that ~250,000 open fractures involving VML occur annually in the US. Furthermore, 92% of the muscle injuries were identified as VML in a cohort of battlefield-injured military personnel [4]. The injured area in VML is so severely compromised that the muscle loses its innate capacity to heal the wound, leading to chronic functional deficits, poor muscle strength, and life-long disability and dysfunction [5]. Surgical interventions are, therefore, mandatory to treat such massive injuries. 

Current surgical approaches mostly use autologous grafts and acellularized scaffolds, which can impede a patient’s successful recovery [3,6]. For instance, in autologous tissue transfer, donor site morbidity is prevalent, leaving impaired integration of the implanted muscle with the neighboring musculature [7]. Furthermore, autologous grafts can exhibit poor mechanical properties, and in many cases, it becomes difficult to obtain good-quality autologous grafts [6]. On the other hand, pathologic fibrotic response is expected in synthetic acellular scaffold treatment, leading to dysfunctional muscle formation and compromised functional recovery [8,9]. Furthermore, it is still challenging to recapitulate the complexity of muscles with conventional fabrication methods [10].

New therapeutic approaches are required that will promote de novo regeneration of skeletal muscle fibers, integrate with the remaining healthy musculature, and restore muscle strength and functionality. To this end, new studies are focusing on utilizing bioprinting to develop cell-containing scaffolds with greater bioactive and tissue regenerative response. Notably, 3D bioprinting is a highly efficient biofabrication technique that can be used to develop design-specific or defect-specific cell-laden scaffolds or constructs [11]. There is ample evidence that 3D bioprinting can be used to develop “live” tissues and organs by using bioinks that contain cultured cells while keeping more precise geometries that mimic anatomical structures [12]. Such 3D bioprinting has been effectively utilized in skeletal muscle tissue engineering with promising results over the past few years [13]. Recent advancements have also led to improving bioprinting strategies to construct large muscle structures, promote myogenesis and enhance skeletal muscle tissue integration [14]. 

One of the most critical aspects of bioprinting scaffolds or constructs is identifying the optimum hydrogel-based inks with the desired characteristics so that they can be efficiently utilized in a specific bioprinting fabrication technique [15]. Generally, such inks should be biocompatible, bioactive, viscoelastic, permeable to oxygen and nutrients, and mimic the target tissue’s extracellular matrix (ECM), thus creating an environment where the cells can survive, proliferate, and differentiate. The inks should also exhibit optimum in situ gelation kinetics so that the bioprinted products exhibit favorable structural fidelity and stability post-printing. In addition, the choice of the ink depends on the printing modality. For instance, low-viscosity ink is used for inkjet bioprinting to avoid clogging [16]. On the other hand, inks with higher viscosity and shear thinning properties are essential in extrusion bioprinting to avoid shear force damage to the cells [17]. However, inks with excessively high viscosity can limit cell spreading, migration, proliferation, and matrix remodeling. 

Over the past few years, extensive research in bioprinting has led to the development of several kinds of hydrogel inks, commonly known as bioinks [17], some of which have even been used to treat skeletal muscle defects. Among them, alginate-based cell-laden bioinks are quite common. Alginate is a biodegradable and biocompatible polysaccharide extracted from brown algae, which can be gelated when ionically crosslinked. It has been employed to print vascular tissue, bone, and cartilage mimics [18]. Alginate is also commonly utilized to develop bioinks for cell-laden bioprinting skeletal muscle constructs [19]. For instance, alginate/poly(ethylene glycol)-fibrinogen bioinks or gelatin methacryloyl-alginate bioinks containing C2C12 muscle cells were developed for functional skeletal muscle tissue replacement [20,21]. 

Gelatin is another well-explored biomaterial to develop bioinks, primarily as gelatin methacrylate (GelMA). Typically, alginate and/or GelMA are mixed with other biomaterials to develop composite bioinks or combined to make alginate-GelMA composite bioinks. However, little effort has been made to develop alginate–gelatin composite bioinks for skeletal muscle tissue engineering applications. While alginate lacks the desired bioactive properties and gelatin exhibits poor mechanical strength, optimum formulations of their combination could lead to bioinks suitable for bioprinting skeletal muscle tissue scaffolds with the desired bioactive and material properties [22]. Gelatin can provide RGD cell adhesion motifs in the alginate–gelatin bioinks and instill bioactivity and cell adhesion capabilities in the alginate bioinks [23]. Prior studies have indicated that oligopeptide sequences containing RGD peptide sequences result in better C2C12 myoblast cell adhesion and optimum scaffold degradation than pure alginate [24,25]. Recently, extrusion-bioprinted alginate–gelatin bioinks enabled C2C12 cells to grow in the direction of printing, migrate to the hydrogel surface over time, and differentiate into ordered myotube segments [26]. Taken together, alginate–gelatin composite bioinks offer the potential for developing bioprinted scaffolds to treat VML defects. 

In this study, we developed alginate–gelatin hydrogel inks to be potentially used as bioinks and utilized them in an extrusion-based bioprinter to develop bioactive and biodegradable scaffolds suitable for treating skeletal muscle defects. We optimized the ink formulation and printing parameters, as this was essential to develop high-resolution scaffolds with favorable structural fidelity. In addition, we explored the effect of crosslinking on the physicochemical and mechanical properties of the scaffolds and established the relation between crosslinking parameters and the scaffold characteristics (stiffness, swelling, and degradation) such that they are suitable for durable skeletal muscle tissue constructs. We believe that this is one of the first studies to perform a detailed processing-structure-property analysis of alginate–gelatin compositions for extrusion bioprinting of skeletal muscle tissue constructs. 

## 2. Materials and Methods

### 2.1. Materials

Sodium alginate, gelatin (from porcine skin, Gel Strength 300, Type A), and calcium chloride (CaCl_2_) were procured from Sigma-Aldrich (St Louis, MO, USA) and used without modifications. 

### 2.2. Hydrogel Ink Development

First, sodium alginate powders were dissolved in 1× phosphate-buffered saline (PBS) and stirred with a magnetic stirrer for 1 h at 80 °C until completely dissolved. Alginate concentrations were varied at 4%, 6%, 8%, and 12% *w*/*v*. In a separate beaker, gelatin powders were mixed in 1× PBS for 30 min at 60 °C. Gelatin concentration was kept fixed at 6% *w*/*v*. After dissolving the individual gelatin and alginate solutions, the gelatin solution was added to the alginate solution under slow stirring at 1:1 *v/v* and mixed for 1 h at 60 °C to ensure homogeneity. The composite alginate–gelatin hydrogel inks were left on a hot plate to achieve a temperature of 37 °C before printing. Stock solutions of the inks were stored at 4 °C for further usage. The specimen names are mentioned in Table 1.

### 2.3. Extrusion Printing of the Scaffolds

#### 2.3.1. Ink Evaluation: Manual Dispensing

Before printing the scaffolds in a bioprinter, we evaluated the extrudability of the composite inks by the manual dispensing method. The composite boinks maintained at 37 °C were transferred into 3 mL pneumatic cartridges (CellInk, Boston, MA, USA) equipped with a 27 G clear nozzle at the tip of the cartridge. If the composite ink successfully extruded out of the nozzle with normal hand pressure (and without much force), forming a continuous fiber, it was considered to be suitable for printing. 

#### 2.3.2. Optimization of Extrusion (Bio)Printing Parameters

After validating the inks with the manual dispensing method, the cartridges were loaded onto the BioX bioprinter (CellInk, Boston, MA, USA). Wevaried extrusion printing parameters such as print pressure (10–30 kPa), printing speed (15–30 mm/s), and layer height (0.1–0.2 mm) and analyzed their effect on the print resolution of the scaffolds (20 × 20 × 1 mm^3^) with a honeycomb infill pattern. The infill density was fixed at 25%. Furthermore, the print bed and nozle temperatures were fixed at 25 °C and 37 °C, respectively, at all times. The print bed temperature acted as a thermal crosslinker for gelatin in the alginate–gelatin scaffolds. First, we qualitatively analyzed the scaffolds and graded them on a scale of 1–5, with 1 being the worst, and 5 being the best. The printing parameters that resulted in scaffolds with scores above 3.5 was determined suitable for printing the scaffolds for further quantitative analysis. However, once the combination of optimum printing parameters was determined, they were used to print the scaffolds (20 × 20 × 1 mm^3^) out of the various alginate-gelatin inks. 

### 2.4. Rheological Analysis

The alginate–gelatin inks were analyzed using a Physica MCR 301 Rheometer (Anton Paar, Ashland, VA, USA). All experiments were performed using a parallel plate fixture (PP50, diameter 50 mm diameter, 0.2 mm gap) at 37 ± 0.1 °C. Amplitude sweep tests were first obtained to determine the linear viscoelastic region (LVR) from the elastic (storage; G′) and viscous (loss; G″) moduli vs. strain (γ) plots, at a constant angular frequency (ω = 10 rad/s) with 0.1–1000% strain. Frequency sweeps were obtained in the LVE region, at a constant 1% strain amplitude, over a frequency range of 0.1–100 rad/s. To determine the flow properties of samples, a concentric cylinder measuring system (CC27, 26.66 mm diameter) was used, and the viscosity (η, Pa.s) was obtained by setting shear rate ($\dot{\gamma }$) within 0.01–1000 s^−1^ range. Shear stress (σ) vs. shear rate ($\dot{\gamma }$) curves were evaluated to determine the flow behavior of the inks. 

### 2.5. Print Accuracy

It is essential to obtain a high degree of print accuracy and develop high-resolution printed scaffolds. First, we qualitatively analyzed the scaffolds and graded them on a scale of 1–5, with 1 being the worst and 5 being the best. Scaffolds scoring below 3.5 were deemed unworthy for the quantitative print accuracy analyses. For quantitative analyses, scaffolds (20 × 20 × 1 mm^3^) were printed based on varying alginate–gelatin composite inks and imaged using a digital camera. The digital images were then analyzed in ImageJ (National Institute of Health, Bethesda, Maryland, MD, USA) and the print accuracy *P* was calculated using the equation $P=1-\frac{\left|a-b\right|}{b}\times 100$, where *a* is the printed area of the scaffold, and *b* is the design area of the printed scaffold [27]. 

### 2.6. Crosslinking Effect

Crosslinking is mandatory to stiffen the hydrogel-based printed scaffolds for easy handling of the scaffolds. Various calcium chloride (CaCl_2_) concentrations and crosslinking durations were explored to determine the most optimized crosslinking parameters suitable for the constructs. After printing, the scaffolds were immersed in CaCl_2_ solution (300 mM, 400 mM, or 500 mM) for varying durations (5 min,10 min, or 15 min). After crosslinking, samples were retrieved from the CaCl_2_ bath and patted dry using Kimwipes. The stiffness of the crosslinked samples was then analyzed on a universal testing machine (UTM), as mentioned in the following section. 

### 2.7. Mechanical Properties Analysis of the Scaffolds

The mechanical properties, i.e., stiffness of the scaffolds, were investigated using compression testing on a UTM. Cylindrical scaffolds with dimensions Ø 20 mm × 4 mm were printed using extrusion (bio)printing and placed between the flat compression plates of the UTM The upper and lower sample surfaces were fixed to the platens of the UTM machine to ensure that there was no slippage during testing. Before the actual measurement, the samples underwent ten preconditioning cycles to a strain of 10% at a rate of 0.1 mm/s to ensure that the structure of the samples was at a consistent and repeatable reference state [27,28]. Subsequently, a loading rate of 0.1 mm/sec with a 10 N load cell was applied to the specimens, and the test continued until the specimens cracked or got squished entirely. Subsequently, the stiffness of the scaffolds was determined as the slope of the linear region of the stress-strain curve [27,28].

### 2.8. Swelling Analysis

The printed scaffolds were first crosslinked in 300, 400 or 500 mM CaCl_2_ for 15 min. Fifteen minutes was chosen as the crosslinking time for the swelling and degradation analysis as it yielded scaffolds with optimum stiffness. After crosslinking, the samples were patted dry with kimwipes and weighed as *W_i_*. Subsequently, they were immersed in Dulbecco’s Modified Eagle Medium (DMEM) (Thermo Fisher Scientific, Waltham, MA, USA) at 37 °C for varying durations (3, 7, or 14 days). At the end of each time point, samples were retrieved, carefully patted dry with kimwipes, and weighed as *W_f_*. The swelling extent *S* was calculated using the equation $S=\frac{W_{f}-W_{i}}{W_{i}}\times 100$.

### 2.9. Degradation Analysis

The printed scaffolds were crosslinked in 300, 400 or 500 mM CaCl_2_ for 15 min, dried in a desiccator, and then freeze-dried for 24 h. Subsequently, the initial dry weights (*W_d_*) of the samples were recorded. The scaffolds were then immersed in DMEM at 37 °C for varying durations (3, 7, or 14 days). At the end of the time points, samples were retrieved, dried in a desiccator, and freeze-dried for 24 h. Post freeze drying, the scaffolds were weighed and recorded as *W_f_*. The degradation percentage D was calculated using the equation $D=\frac{W_{d}-W_{f}}{W_{d}}\times 100$.

### 2.10. Biocompatibility Analysis

The printed scaffolds of different alginate–gelatin compositions were crosslinked at 500 mM for 15 min and then sterilized by immersing them in 90% ethanol and exposing it to UV. Subsequently, the scaffolds were placed in wells filled with DMEM for 4 h. Then C2C12 muscle cells were seeded on the scaffolds, and it was ensured that the scaffolds stayed submerged in DMEM supplemented with fetal bovine serum. After 3 or 7 days of culture, the scaffolds were retrieved, rinsed three times in PBS, and prepared for the assay. First, Thiazolyl blue tetrazolium bromide (MTT, Sigma Aldrich, Burlington, MA, USA) stock solution was added to the specimens, followed by 4 h incubation at 37 °C. Then, dimethyl sulfoxide (DMSO) was used to dissolve formazan, and finally, OD570 readings were recorded using a spectrophotometer. 

### 2.11. Statistical Analysis

All tests were carried out in triplicate. One- and two-way Analysis of Variance (ANOVA) and post-hoc Tukey’s test were performed to analyze the data. A level of significance of *p* < 0.05 was chosen for all the statistical analyses. 

## 3. Results and Discussion

### 3.1. Printability of the Alginate–Gelatin Inks and Qualitative Assessment of Scaffolds

**Figure 1:**
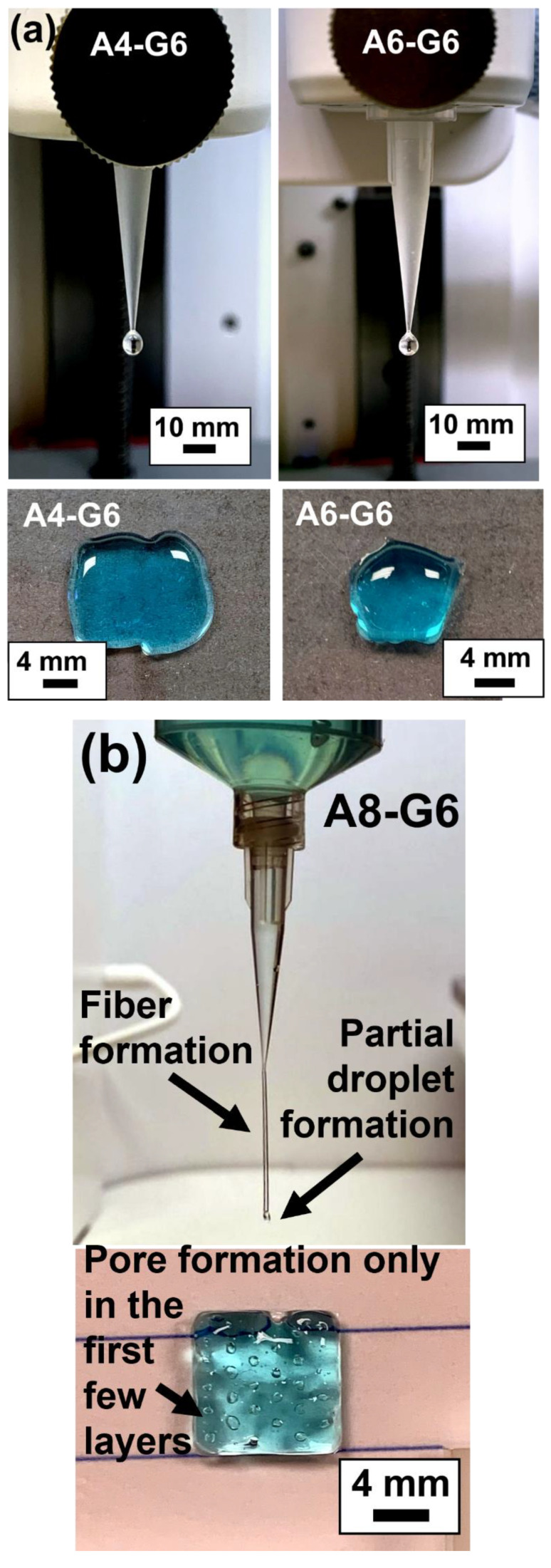
Manual dispensing results of the inks and the corresponding scaffolds printed with the same composition. (**a**) A4-G6 and A6-G6 inks predominantly formed droplets when extruded out of the nozzle. The printed scaffolds exhibited no structural and shape fidelity. (**b**) A8-G6 inks formed partial fiber formation and droplets. The scaffolds exhibited satisfactory shape and structural fidelity. However, pores were only formed in the first few layers.

We performed a preliminary evaluation using the manual dispensing method to validate the ink extrudability [29]. Manual dispensing is a simple yet reliable initial assessment method that indicates whether the ink can form fibers rather than droplets, i.e., whether it is able to form high-resolution scaffolds with good structural fidelity. Specifically, this testing validates the capability of inks to fulfill two critical requirements for extrusion bioprinting. First, the inks should form consistent, cylindrical fibers. Second, the ink should be able to stack layers into coherent structures [29]. Figure 1a,b show representative images from manual dispensing of various alginate–gelatin ink formulations and the corresponding scaffolds that were printed using the inks. When dispensed through the bioprinting nozzle attached to the syringe, the 4% and 6% alginate ink concentrations formed droplets, not continuous fibers. The corresponding structure images of the scaffolds further demonstrate the printability of the inks and their suitability for printing scaffolds.

The images of the printed structures corroborate the dispensing results. For instance, A4-G6 and A6-G6 ink could not be used to print scaffolds with definite structures. Specifically, the A4-G6 ink exhibited a liquefied nature, perhaps due to low viscosity; hence, it spread out on the print bed plate after printing and was unable to form the scaffold of the pre-determined design. The printed scaffold was also liquefied in nature and did not retain its structural fidelity once it had been deposited on the bedplate. The A6-G6 ink exhibited relatively higher viscosity than the A4-G6 and therefore did not spread out on the bed plate. However, it could not form a scaffold with proper outlines or the designed pores. In both cases, the cooling effect of the bedplate served as a thermal crosslink for the gelatin and gelated the ink. This helped control the ink’s liquefied nature and to retain the scaffold’s intended structure.

The A8-G6 ink exhibited partial droplet and partial fiber formation (Figure 1b), which indicated that it was suitable for bioprinting but not optimal for the creation of a high-resolution scaffold. The printed structure retained its shape, and proper outlines were also observed, but the designed pores were not formed. We specifically chose a porous scaffold design to be printed in order to analyze the inks’ ability to create pores. Unfortunately, none of the 4, 6, and 8 vol.% alginate concentration inks were able to form the pores because of their low viscosity and diluted nature. We also explored different printing parameters to utilize these inks for printing the scaffolds with acceptable print resolution and structural fidelity during the minimum window required to apply the crosslinking agent. Yet, as shown in Table 2, Table 3, Table 4 and Table 5, none of these inks was suitable for printing the scaffolds.

**Figure 2:**
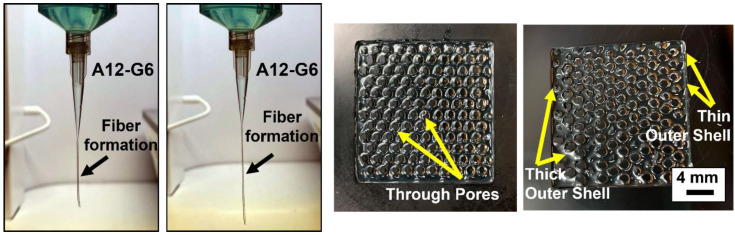
Manual dispensing results of A12-G6 bioink and the corresponding scaffolds printed using the same composition. The scaffolds exhibited high structural and shape fidelity. Moreover, they exhibited high resolution with the formation of through and well-defined pores.

In contrast, the A12-G6 ink formed coherent fibers instead of droplets (Figure 2), indicating that this ink would be suitable for printing structures with good shape, structural fidelity, and high-resolution [29]. The corresponding digital images in Figure 2 demonstrate the high print resolution of the structures with non-clogged and through pores, detailed pore outlines, and well-defined structure boundaries, with either thick or thin outer shells.

Prior studies suggested that 2–5 vol.% alginate is suitable for printing scaffolds [22,30,31,32,33]. Even though the final alginate concentration in the ink depends on the printing technique, alginate concentrations of less than 6% *w*/*v* were generally deemed suitable for developing constructs with good shape and structural fidelity. However, in such cases, alginate was compounded with additional natural biomaterials that helped increase the viscosity of the ink. For instance, the addition of 9% methylcellulose to 3 wt.% alginate notably enhanced the ink viscosity compared to pristine alginate inks, resulting in precise deposition of the ink to develop the scaffolds [34]. Similarly, when 2.25% *w*/*v* nanofibrillated cellulose was added to 0.25% *w*/*v* alginate, it drastically increased the shape fidelity of the printed scaffolds compared to only 3% *w*/*v* alginate scaffolds [35]. Moreover, synthetic polymers such as poly(acrylamide) [36] and poly-lactic acid (PLA) continuous fibers [37] have been incorporated into alginate inks to increase the printability of the bionks and develop scaffolds with high structural and shape fidelity along with good mechanical properties. 

We could only achieve acceptable printability results with 12% *w/v* alginate in the present study. This was primarily because alginate was not compounded with other materials that could strengthen the ink and provide additional structural fidelity to the printed scaffolds. In the present case, alginate was compounded only with gelatin solely to increase the bioactivity of the composite. We chose to form alginate–gelatin composites to retain the flexibility of the scaffolds that are critical for skeletal myogenesis and myotube formation in skeletal muscle tissue engineering [38]. In certain instances, the alginate inks were crosslinked before printing, as performed in other studies [29], in order to stiffen the ink leading to a stable structure. Another strategy is to incorporate additional materials such as GelMA into the alginate ink in order to provide an extra crosslinking step (such as photo-crosslinking), resulting in added structural fidelity to the constructs [21]. 

### 3.2. Bioprinting Parameters

The pneumatic or printing pressure and speed were identified as the most important parameters influencing the overall print resolution of the scaffolds. Optimizing the viscosity of the alginate–gelatin ink formulations is essential to obtain printed scaffolds with high structural fidelity; however, the airflow pressure and print speed are significant factors influencing the print accuracy and resolution of the printed scaffolds. From the data shown in Table 2, Table 3, Table 4 and Table 5**,** the optimum printing pressure and speed depend on the kind of ink used. For instance, a lower printing pressure and speed is recommended for developing scaffolds with lower alginate concentrations (<8% *w*/*v*). However, higher printing pressure, such as 30 kPa, and lower printing speed, such as 15 mm/s print speed were identified as the optimum parameters for developing A12-G6 scaffolds. The printing pressure is a critical factor in extrusion bioprinting as high extrusion pressures can induce high shear in the ink and hamper the cells contained within. Hence, optimizing the print (extrusion) pressure is advisable so that the ink easily flows out of the nozzle at a rate capable of creating fine strands and, eventually, a high-resolution scaffold without creating excessive stresses to hamper the cell viability. Additionally, narrow extrusion nozzles (250 µm Ø) and increased extrusion pressure (60 kPa) can be beneficial in developing scaffolds with a specific orientation and orienting muscle cells to form directional myotubes [26]. 

Print speed is an essential factor in 3D printing, as it influences the resolution of the scaffolds [39,40]. Previous efforts have also achieved higher resolution accuracy with printing speeds of 60 mm/s [21] and 50 mm/s [41]. However, most of these parameters depend on the viscosity of the ink. For example, in the present study, much lower working ranges of the printing pressure and speeds were used for the other inks as compared to the A12-G6 inks, primarily because inks with less than 12% *w*/*v* alginate exhibited much lower viscosity and could easily be extruded. As opposed, A12-G6 inks exhibited the highest viscosity and required much more force to be extruded. Moreover, an optimum printing speed helps deposit thin filaments (around 1.5 mm) with precision [41], thus helping obtain a high resolution for the scaffolds. 

### 3.3. Rheological Properties of the Alginate–Gelatin Inks

**Figure 3:**
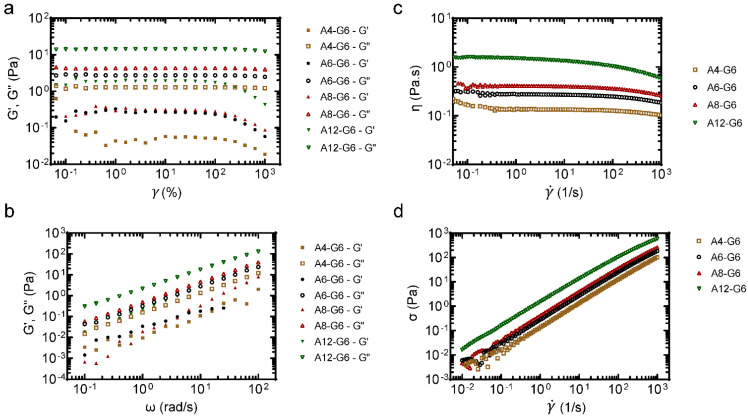
Rheological analysis of the inks. (**a**) Amplitude sweep test (Elastic modulus (G′), storage modulus (G′′) vs. strain (%)). (**b**) Frequency sweep test (Elastic modulus (G′), storage modulus (G′′) vs. angular frequency (ω (rad/s)). (**c**) Viscosity (Pa.s) was plotted as a function of shear rate ($\dot{\gamma }$ (1/s)). (**d**) Evaluation of flow behavior with shear stress (s) vs. shear rate ($\dot{\gamma }$) curves.

Polymer-based composites commonly exhibit viscoelastic behavior, which is related to their molecular structure and formulation. Evaluating the relationship between molecular structure and viscoelastic behavior requires that rheological measurements be conducted in regions where the viscoelastic properties are independent of imposed strain values, i.e., LVR. Hydrogels made of gelatin and alginate offer a wide range of flexibility in final properties as gelatin is more solid-like while alginate is more liquid-like in nature. The viscoelastic characteristics of the inks were obtained from the amplitude sweep and frequency sweep tests. Representative amplitude sweeps (LVR) of four inks with varying alginate concentrations are shown in Figure 3a. It can be noted that both G′ and G′′ increased with increasing alginate content, and the inks were viscous (i.e., G′′ > G′), which might be due to the entanglement network between the alginate and gelatin biopolymers. Figure 3b shows representative frequency sweeps of the inks. Notably, the G′′ was always significantly higher than G′ (*p* < 0.05) for each gel type, and they both increased linearly with increasing ω (*p* < 0.05). The gels exhibited viscous behavior similar to that reported by others [31,42,43].

The flow curves (Figure 3c) show that: (i) the gel viscosity increased with increasing alginate concentration from 4–12%, presumably due to the stronger network structure and higher resistance to flow; and (ii) the viscosity peaked at $\dot{\gamma }$ = 0.1 s^−1^ in all cases, after which it slightly decreased with increasing $\dot{\gamma }$, indicative of typical non-Newtonian shear-thinning behavior. Typically, higher viscosities at low shear rates indicate 3D structure formation before shearing. With increasing shear rate, such 3D hydrogel structures were broken down, and the fluid trapped inside was released. This led to shear-thinning and reduced gel viscosity with increasing shear rate, at all alginate concentrations, albeit more pronounced at higher alginate concentrations. Since water’s viscosity is slightly less than 10^−3^ Pa s at 37 °C, these gels were at least 2–3 orders of magnitude more viscous than water [44]. 

The flow curves data were first fitted to a simple power-law relationship given by the equation: $\eta \left(\dot{\gamma }\right)=m{\dot{\gamma }}^{\left(n-1\right)}$. Here, *m* is the consistency index which describes the magnitude of *η*, and *n* is a dimensionless parameter describing the response in viscosity to increasing $\dot{\gamma }$. While *n* = 1 for Newtonian fluids, *n* > 1 for shear-thickening, and *n* < 1 for shear-thinning behaviors. The parameters from the power-law model fit are reported in Table 6. It was noted that *n* is slightly less than one in all cases, indicative of shear-thinning behavior. While no noticeable trend in *n* values in relation to gelatin lading (*p* > 0.1) was noted, *m* increased with gelatin loading in agreement with the graphical observations (*p* < 0.01). However, power-law or Carreau-Yasuda models have limited predictive power despite their simplicity, flexibility, and wide usage.

Figure 3d shows the shear stress experienced by the inks with different shear rates. It can be observed that the shear stress increased linearly with an increasing shear rate for all inks. Additionally, the shear stress increased with increasing alginate levels at a given shear rate. A simple Herschel–Bulkley model ($\tau =\tau_{0}+k\gamma^{n}$) could be used to fit the flow behaviors of such solutions, where *τ* is the shear stress, *τ*_0_ is the yield stress, γ is the shear rate, *k* is the consistency coefficient, and *n* is the dimensionless flow index. This model fitted well (*R^2^* > 0.99) in all cases (Table 6), and the fitted *k* values (representing apparent viscosities of the solutions) were consistent with the measured apparent viscosity data (Figure 3c).

In uncrosslinked viscoelastic liquids, it is normal to observe *G*′′ > *G*′, as the bonds are weak between the biopolymer chains. It should be noted that the viscoelastic properties of gelatin-alginate solutions presented here (i.e., *G*′ < *G*′′, tan δ > 1) stand in contrast to reports by others. This could primarily be due to the differences in the ratio of gelatin to alginate. For instance, Mondal et al. [31] developed hydrogels with fixed 4% gelatin and varying alginate levels (3–4%) and noted that these gels exhibited *G*′ > *G*′′ (tan δ < 1) and shear-thinning behavior and the hydrogel strength increased with increasing alginate loading.

### 3.4. Quantitative Assessment of Structures–Print Accuracy

**Figure 4:**
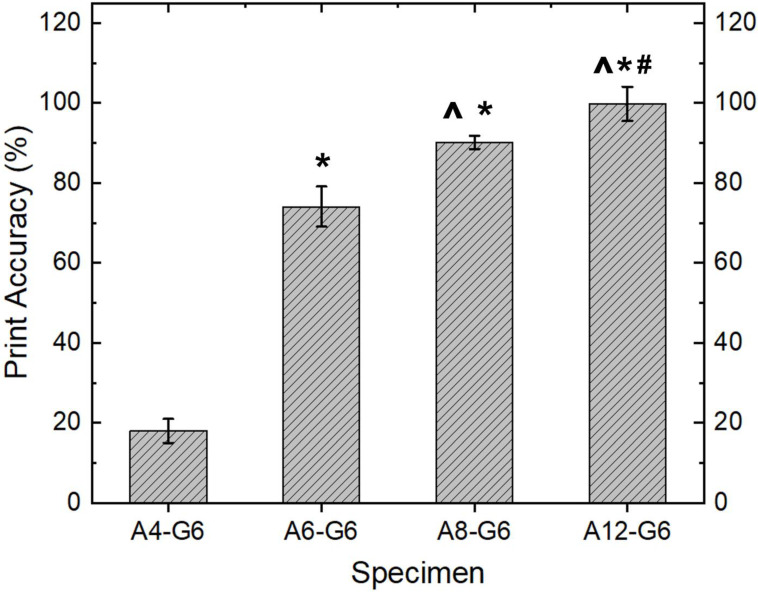
Print accuracy of the varying printed constructs. * indicates *p* < 0.05 compared to A4-G6. **^** indicates *p* < 0.05 compared to A6-G6. **#** indicates *p* < 0.05 compared to A8-G6.

In addition to the manual dispensing results and the qualitative evaluation of the scaffolds, it is essential to quantitatively analyze the quality of the printed part with the respective inks. Therefore, the print accuracy test was performed to analyze the resolution of the printed scaffolds using various inks (Figure 4). Overall, a higher alginate concentration significantly (*p* < 0.05) increased the resolution and print accuracy of the printed scaffolds, whereas lower alginate concentrations resulted in scaffolds with poor print accuracy and resolution. For instance, results show a low printing accuracy (18%) in the case of the A4-G6 scaffolds. The latter result is on par with the qualitative evaluation of the A4-G6 ink, indicating a poor resolution of the A4-G6 scaffolds. In contrast, compared to the A4-G6 scaffolds, the A6-G6 and A8-G6 inks resulted in structures with high print accuracy, 72 and 91%, respectively. However, even though the A6-G6 and A8-G6 printed scaffolds were within the design dimensions, they were not suitable for making high-resolution scaffolds with well-defined outlines and pore geometry. Hence, the shape and structural fidelity of the scaffolds should not be solely analyzed based on quantitative print accuracy assessments alone, they should always be supplemented with qualitative analysis. Comparable with the manual dispensing and qualitative analysis results, A12-G6 yielded scaffolds with the highest print accuracy (99.94%). Also, the scaffolds exhibited good shape and structural fidelity for at least 15 min or till the crosslinking solution was applied.

The structural uniformity of a hydrogel is critical for obtaining a uniform cell distribution within the scaffold. In addition, scaffolds with good structural fidelity exhibit uniform mechanical properties (stiffness) and controlled degradation kinetics. Generally, it is challenging to print scaffolds with good structural fidelity using alginate-based inks due to alginate’s inherently low viscosity. However, higher alginate concentrations can mitigate the latter issue. In the present case, 12% w/v alginate helped develop the scaffolds with high print accuracy and resolution and good structural fidelity. Similarly, Luo et al. [45] developed highly concentrated alginate/polyvinyl alcohol bioinks (>12.5–18.2 wt.% alginate) for extrusion bioprinting and developed highly stable uncrosslinked structures. It should also be noted that many studies in the literature observed varying printability and structure resolution with different alginate concentrations. For example, Ojansivu et al. [46] developed bioinks containing 5% gelatin and 4% alginate and printed good-quality structures, while we could only print unstable liquefied structures with a similar composition. On the other hand, Othman et al. [41] could only achieve high-fidelity structures with alginate 10% and gelatin 50%. However, the bioinks were too viscous to be printed after some time, and the scaffolds were soft. This might be due to the differences in the molecular weight of the feedstock alginate. Along those lines, the molecular weight of alginate can also be tweaked to enhance the printability of the bioink and develop structures with good structural fidelity [47].

### 3.5. Effect of Crosslinking on the Printed Scaffolds

**Figure 5:**
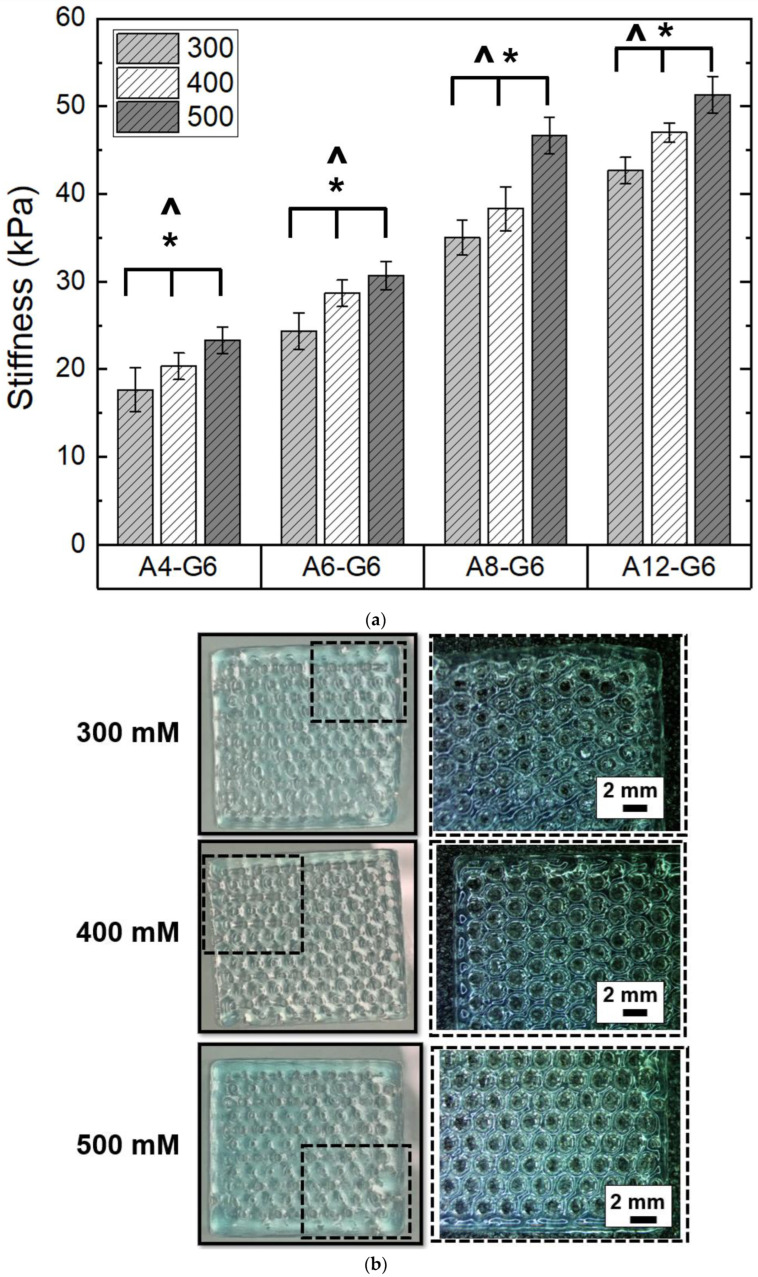
Effect of crosslinking concentration on the stiffness of the printed scaffolds. (**a**) Stiffness of the constructs when crosslinked with different calcium chloride (CaCl_2_) concentrations and 15 min of crosslinking time. * indicates that at 0.05 level (two-way ANOVA), the mean stiffness of the scaffolds crosslinked with different crosslinking concentrations is significantly different. **^** indicates that at the 0.05 level (two-way ANOVA), the mean stiffness of the scaffolds with different alginate concentrations is significantly different. (**b**) Digital images of the scaffolds (20 × 20 × 1 mm^3^) indicate the effect of varying CaCl_2_ concentrations on the printed scaffolds. The corresponding stereomicroscopy images show the magnified view of the dotted regions in the scaffolds.

We first varied CaCl_2_ concentrations and analyzed their effect on the extent of crosslinking of the alginate–gelatin scaffolds. The measuring criteria for analyzing the crosslinking extent was the stiffness/flexibility of the scaffolds, primarily because we wanted to mimic the stiffness of skeletal muscle in the scaffolds. Figure 5a shows the relative stiffness of the alginate–gelatin scaffolds crosslinked with varying CaCl_2_ concentrations. Higher CaCl_2_ concentration significantly (*p* < 0.05) increased the crosslinking effect and stiffness of all the alginate–gelatin scaffolds. However, the crosslinking treatment was ineffective in favorably stiffening the A4-G6 and A6-G6 scaffolds. Both the scaffolds exhibited low stiffness and an overall squashy texture after crosslinking; moreover, only the outer surfaces of the scaffolds coagulated, while the inner core of the scaffolds remained notably squashy. This is because the CaCl_2_ solution could not percolate through the scaffold surfaces effectively. In addition, the absence of the pores significantly inhibited the CaCl_2_ solution from being in contact with the inner surface area of the scaffolds. In contrast, the A8-G6 scaffolds exhibited considerably higher stiffness (*p* < 0.05) than the A4-G6 and A6-G6 scaffolds. In addition, the outer surfaces of the scaffolds stiffened, while the inner core was relatively less stiff with a predominant spongy texture. Some pores at the bottom of the scaffold helped the CaCl_2_ solution to percolate through and crosslink the inner core of the scaffold. However, these were not through pores and were absent on the top layers of the scaffold, minimizing the chances of CaCl_2_ infiltration and leaving the inner core spongy.

The A12-G6 scaffolds, on the other hand, exhibited significantly higher stiffness than all the other scaffolds at each CaCl_2_ concentration tested (Figure 5a); notably, the stiffness was uniform over the entire scaffold volume. The crosslinking effect was the highest due to the high alginate concentration, resulting in a more pronounced ionic crosslinking between the Ca^2+^ ions. In addition, the through pores helped in uniform infiltration of the CaCl_2_ solution in all regions of the scaffolds (including the inner core), which led to a uniform crosslinking reaction and increased stiffness in the overall volume of the scaffold. Moreover, statistical analysis indicated that a higher alginate concentration significantly enhanced (*p* < 0.05) the stiffness of the scaffolds (Figure 5a). 

The digital images and the corresponding stereomicroscope images of the crosslinked A12-G6 scaffolds are shown in Figure 5b. The images indicate that higher CaCl_2_ concentration resulted in scaffolds with well-defined scaffold perimeter and pore outlines. Specifically, the scaffolds crosslinked with 500 mM CaCl_2_ concentrations exhibited higher stiffness and more distinct and well-defined pore outlines than the ones crosslinked with 300 mM. Overall, statistical analysis (Two-way ANOVA; *p* < 0.05) indicated that the differences in crosslinking concentrations and alginate concentrations significantly affected the stiffness of the scaffolds. Finally, we chose 500 mM to be the most suitable CaCl_2_ concentration for crosslinking the scaffolds with suitable stiffness. 

**Figure 6:**
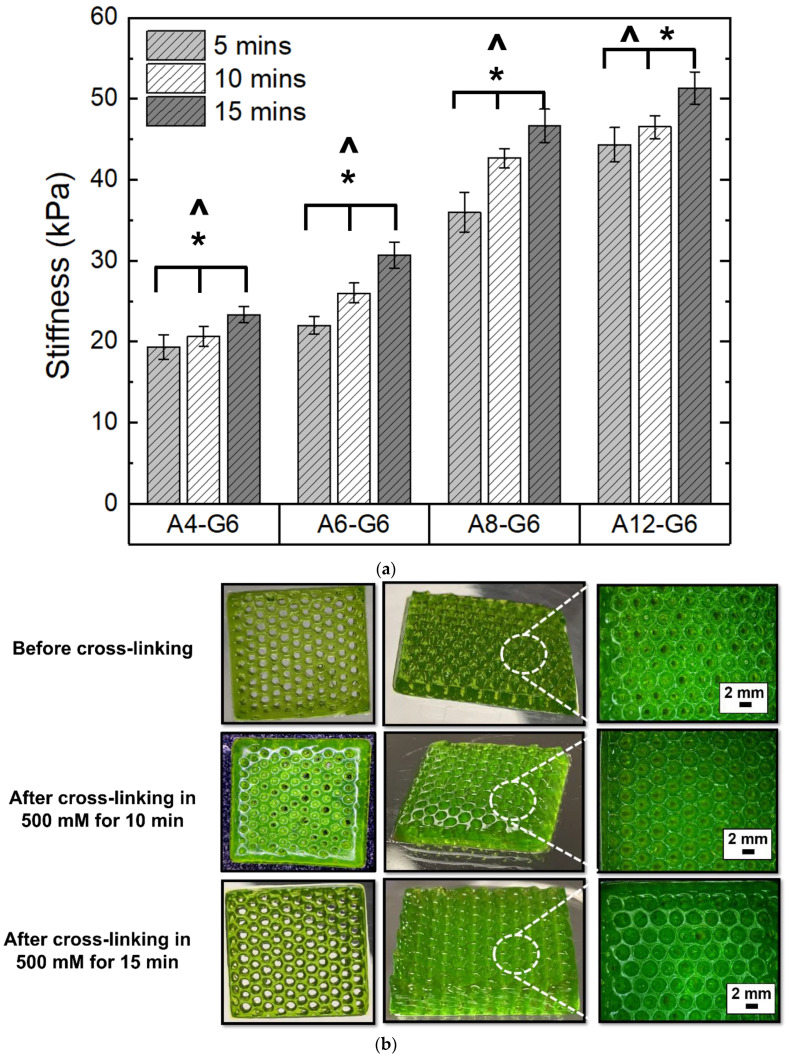
Effect of crosslinking time on the stiffness of the bioprinted scaffolds. (**a**) Structural fidelity and stiffness of the constructs when crosslinked with 500 mM CaCl_2_ for 5, 10, or 15 min. * indicates that at 0.05 level (two-way ANOVA), the mean stiffness of the scaffolds crosslinked with varying crosslinking times is significantly different. **^** indicates that at the 0.05 level (two-way ANOVA), the mean stiffness of the scaffolds with varying alginate concentrations is significantly different. (**b**) Digital images of the scaffolds (20 × 20 × 1 mm^3^) indicate the effect of different crosslinking times on the printed scaffolds.

Figure 6a shows the effect of crosslinking time on the stiffness of the scaffolds with different alginate concentrations. The increase in crosslinking time significantly enhanced (*p* < 0.05) the stiffness of the scaffolds. Figure 6b shows the effect of crosslinking time on the A12-G6 scaffolds. The crosslinked scaffolds exhibited distinct outer boundaries and a crisper morphology than the uncrosslinked ones. Further, as seen in the stereomicroscope images, crosslinking for 10 and 15 min with the CaCl_2_ solution notably increased the resolution of pore boundaries compared to the uncrosslinked ones. However, there was no significant perceivable difference in the resolution of the porous scaffolds crosslinked for 10 or 15 min. Both scaffolds with different crosslinking times exhibited pores with well-defined outlines and precise circular geometry.

There are various strategies to crosslink alginate and gelatin-based scaffolds. Ionic crosslinking is one of the most prominent strategies to crosslink alginate-gelatin-based scaffolds. Moreover, the shear forces in extrusion bioprinting align the molecular chains in the alginate–gelatin composite inks, further crosslinked using two routes. First, after printing, the scaffolds were thermally crosslinked by cooling the gelatin at room temperature. This solidified the printed hydrogel, fixing the oriented hydrogel microstructure and gelatin’s RGD motifs, which might help align the muscle cells in a definite direction [48]. In addition, the Arginine-Glycine-Aspartate (RGD) tripeptide motifs are critical for increasing the adhesion of C2C12s, myoblast fusion, and myotube formation [48]. Second, the CaCl_2_ treatment ionically crosslinked the alginate present in the scaffolds, resulting in aligned molecular chains of alginate-gelatin, which can be instrumental in guiding C2C12 cells to migrate, proliferate and differentiate in a definite orientation and direction, thus forming directional muscle strands. Interestingly, studies that focused on developing GelMA-alginate bioinks did not notice a specific orientation of the C2C12 cells, which might be due to the interference of the GelMA photo-crosslinking with the ionic crosslinking of the oriented alginate hydrogel. In contrast, the imine bond in the alginate–gelatin ink scaffolds featured reversible self-healing hydrogel properties, which can help in the dynamic breaking and formation of molecular chains. For instance, the imine (C=N) bonds may break due to the shear forces during printing but can reconfigure themselves in a specific orientation after printing. Post-printing, the ionic crosslinking by the CaCl_2_ treatment preserved the imine bonds of alginate–gelatin in a particular direction, which may be beneficial for providing directional cues to the muscle cells to differentiate and form unidirectional long myotubes. Moreover, the ionic crosslinking of alginate by the Ca^2+^ ions is primarily responsible for providing structural integrity to the scaffolds. External stimuli such as acoustics can be used to trigger muscle cell alignment [49]. However, in the present study, we developed alginate–gelatin printed scaffolds which provide a simple bioink solution that can provide an inherent directional cue for unidirectional myogenic differentiation and myotube formation, leading to dense muscle fiber alignment and formation.

The ionic strength of the crosslinking medium is a major factor that can influence the mechanical properties of the printed scaffolds, which can affect the behavior of encapsulated or seeded cells. We observed that the crosslinking solution’s ionic strength influenced the stiffness of the scaffold, while the crosslinking time was not a significant factor. One of our aims in this study was to develop a scaffold that would mimic the stiffness of innate skeletal muscle, especially when scaffold stiffness can be primal to influence the adhesion, proliferation, and differentiation of muscle cells. Our results (Figure 5a and Figure 6a) show that we successfully developed alginate–gelatin scaffolds with stiffness values mimicking innate skeletal muscle stiffness, which typically ranges from 30–50 kPa [50]. Notably, the crosslinked A12-G6 scaffolds exhibited stiffness in the range of 45–50 kPa, confirming that they simulate the stiffness of the innate skeletal muscle [51], thus making the scaffolds suitable for the VML treatment [52]. The stiffness of the substrate also plays a major role in influencing the proliferation and differentiation behavior of the muscle cells, which will ultimately help in forming the mature myofibers critical for regenerating functional skeletal muscle [53]. Hence, based on the stiffness properties, we hypothesize that the alginate–gelatin compositions developed in this study will be apt to serve as substrates for promoting myogenic differentiation. Moreover, alginate–gelatin scaffolds can be engineered with tunable stiffness for different tissue engineering applications. For example, Kolan et al. [54] developed human stem cell-laden alginate–gelatin scaffolds with 300 kPa stiffness for bone tissue engineering, which requires much stiffer scaffolds than skeletal muscle tissue engineering. Tuning the alginate and gelatin concentrations in the ink is a common approach to adjusting the scaffold stiffness. However, there are other ways to control scaffold stiffness, such as increasing the ionic strength of the solvent (in which the inks are made) to decrease the stiffness and viscosity of the alginate–gelatin ink and increase swelling and degradation behavior in the printed constructs [55]. 

### 3.6. Swelling of the Scaffolds

**Figure 7:**
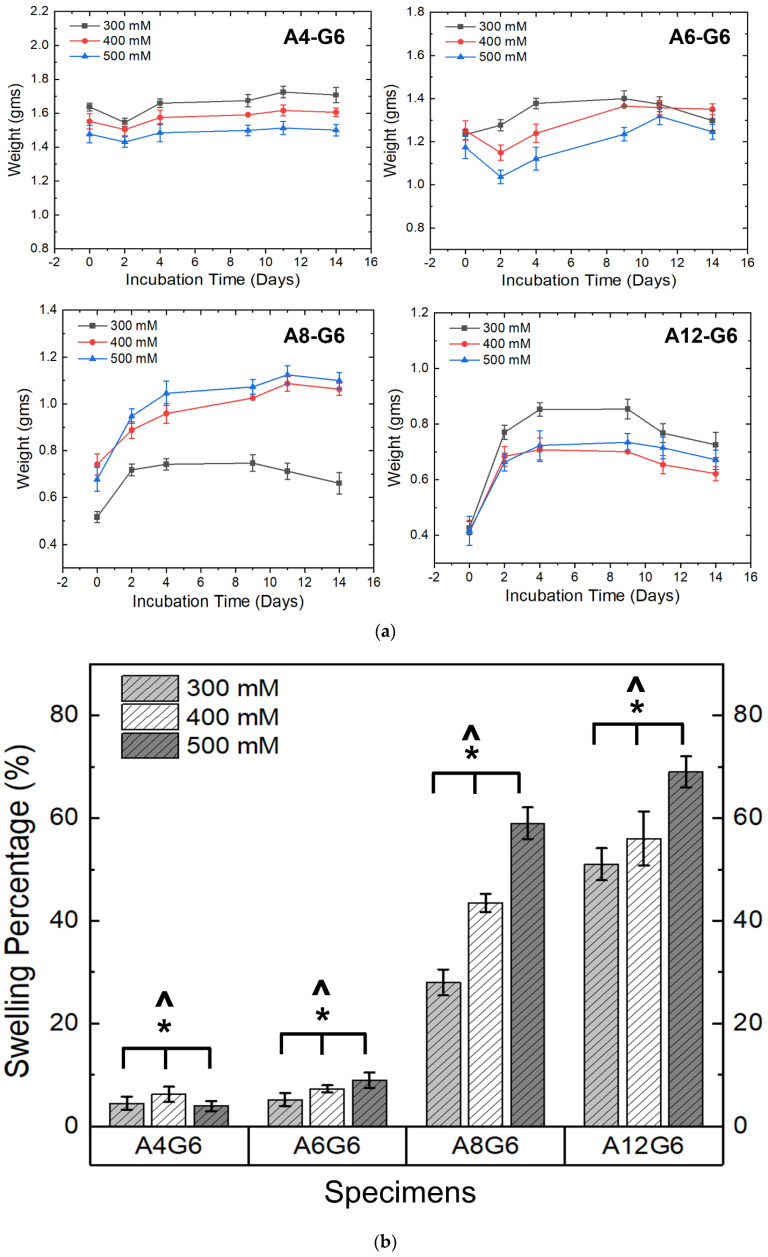
(**a**) Weight distribution of the alginate–gelatin scaffolds over the incubation period indicating the swelling nature of different printed scaffolds over time. (**b**) Swelling percentage of the printed scaffolds over 14 days. * indicates that at 0.05 level (two-way ANOVA), the variation in the crosslinking concentrations had a significant effect on the swelling percentage of the scaffolds. **^** indicates that at the 0.05 level (two-way ANOVA), varying alginate concentrations had a significant effect on the swelling percentage of the scaffolds.

Figure 7a,b show the swelling nature of the various alginate–gelatin scaffolds. The swelling extent of the scaffolds was analyzed by recording their weight increase over time. The A4-G6 scaffolds exhibited a slight decrease in weight for the first 2 days and then increased in weight over the next 12 days. However, the weight increase of the scaffolds was reduced over time (Figure 7a), indicating that they did not exhibit a high swelling percentage at the end of 14 days (2–6%) (Figure 7b). Furthermore, the scaffolds disintegrated into numerous small pieces toward the end of the incubation period, which is a possible reason for the low observed swelling behavior. Similarly, the weight of the A6-G6 scaffolds decreased over 2 days, and the scaffolds did not exhibit significant swelling, but after the second day, they experienced high swelling over the next 8 days. However, after 12 days of incubation, the scaffolds started disintegrating, thereby decreasing the weight, and thus we observed a low overall swelling percentage (5–9%) (Figure 7b). The decrease in weight during the first 2 days may be attributed to the initial degradation of the alginate or the gelatin (mainly gelatin) when immersed in a liquid medium. Nonetheless, once the scaffolds had stabilized in the liquid medium, they absorbed nutrients and water molecules, increasing their weight.

**Figure 8:**
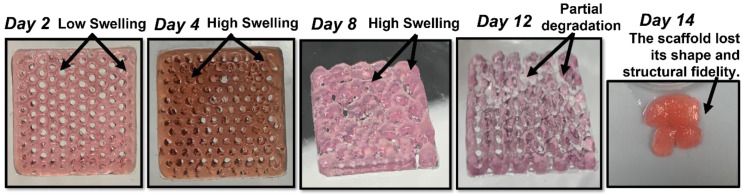
Digital images showing the swelling nature of the A12-G6 printed scaffolds (20 × 20 × 1 mm^3^) over time. The scaffolds were incubated in DMEM at 37 °C over 14 days. The scaffolds first gained weight, but then after 9 days, they started degrading.

Interestingly, the A8-G6 scaffolds crosslinked with different CaCl_2_ concentrations exhibited significant swelling over 14 days. The scaffolds swelling peaked on day 9 or 11. However, a slight decrease in weight could be observed after day 9 or 11, because of the degradation of the alginate and gelatin compounds due to excessive swelling. Additionally, the scaffolds lost their shape and structural fidelity at the end of the incubation period. The A12-G6 scaffolds exhibited a similar nature to the A8-G6 scaffolds, with a swelling percentage of 70% (Figure 7b). The digital images of the incubated scaffolds over time (Figure 8) gave a distinct interpretation of their swelling nature. They experienced a sharp increase in weight, indicating that the scaffolds swelled significantly over the first 2 days, absorbing most of the nutrients, water molecules, and oxygen from the DMEM. The swelling peaked at day 4 and reached a saturation point. It then maintained leveled swelling until day 8 and finally began to degrade, thus decreasing the weight. On day 12, the scaffolds partially disintegrated but retained their shape and structure. However, at the end of the incubation period on day 14, the scaffolds lost their structural fidelity and fell apart.

Notably, the ionic strength of the crosslinking solution significantly (*p* < 0.05) influenced the swelling nature of the scaffold (Figure 7b). A higher ionic strength of the crosslinking concentration increased the swelling percentage of the scaffolds. Hence, scaffolds crosslinked with 500 mM exhibited a higher swelling percentage in the A8-G6 and A12-G6 scaffolds. Moreover, the alginate concentrations also significantly (*p* < 0.05) affected the swelling nature of the scaffolds (Figure 7b). As seen in Figure 7a,b, higher alginate concentrations increased the swelling nature of the scaffolds. Hence, the A12-G6 scaffold crosslinked with 500 mM showed the highest swelling rate (70%). Hydrogels with higher diffusibility of nutrients and oxygen or increased pore formation may counteract cells from migrating to the surface and allow improved 3D cell growth inside the hydrogels. Thus, the A12-G6 ink developed in this study would help achieve high cell densities inside the hydrogels to improve cell (myogenic) differentiation in 3D [26]. 

### 3.7. Degradation Kinetics of the Scaffolds

**Figure 9:**
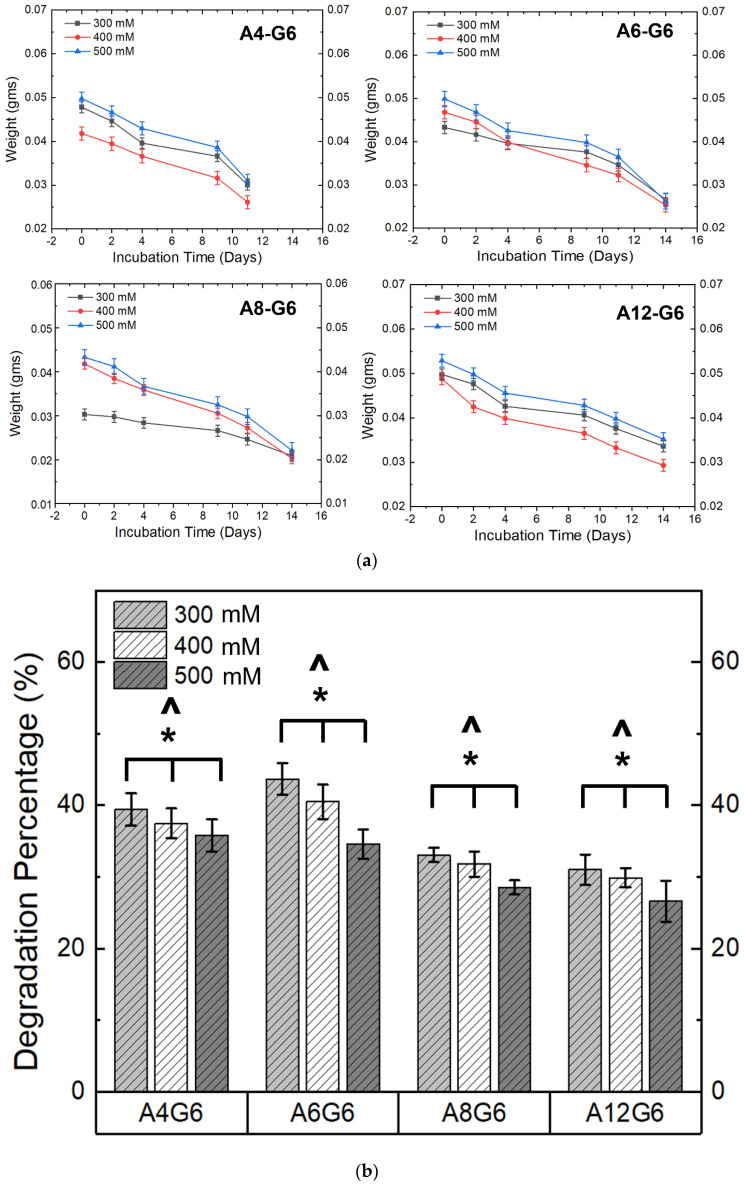
(**a**) Weight distribution of the alginate–gelatin scaffolds over the incubation period indicating the degradation kinetics of different printed scaffolds over time. (**b**) Degradation percentage of the different printed scaffolds over 14 days. * indicates that at 0.05 level (two-way ANOVA), the variation in the crosslinking concentrations had a significant effect on the degradation percentage of the scaffolds. **^** indicates that at the 0.05 level (two-way ANOVA), varying alginate concentrations had a significant effect on the degradation percentage of the scaffolds.

Figure 9a,b show the degradation profile and degradation percentage of the scaffolds. It is evident that the scaffolds treated with different crosslinking concentrations and alginate content underwent degradation in DMEM with varying degradation rates over time. A higher CaCl_2_ crosslinking concentration significantly (*p* < 0.05) reduced the scaffold’s degradation extent (Figure 9b). Moreover, similar to the swelling studies, the alginate content played a major role (*p* < 0.05) in influencing the degradation percentage of the scaffold (Figure 9b). The latter analysis can also be extrapolated from the weight-loss nature of the scaffolds in Figure 9a. For instance, the steep nature of the weight loss indicates that the A4-G6 scaffolds experienced a rapid degradation rate over 12 days. Moreover, the scaffolds disintegrated into numerous small fragments, making it difficult to measure the scaffold at the end of 14 days. Instead, all the other scaffolds, such as the A6-G6, A8-G6, and A12-G6, exhibited a steady degradation profile over time. However, as indicated by the steep weight loss, the degradation rate increased in the A6-G6 and A8-G6 scaffolds toward the end of the incubation period, i.e., after day 11. Notably, compared to other scaffolds, the A12-G6 scaffolds crosslinked with 500 mM experienced a sustained and the least degradation rate over the entire incubation period (Figure 9b), which is critical in long-term tissue engineering scaffolds that might require a prolonged treatment duration for regenerating the native tissue such as the lost skeletal muscle tissue in VML defects.

The sustained degradation of the scaffold resulting in porosity could potentially help the cells to gain more space to proliferate inside the scaffold and achieve enhanced cell density. In addition, the pores could also create a pathway for cells to migrate to the surface, leading to a high cell density and an eventual enhancement in differentiation. However, there is an increased chance for the cells to migrate to the scaffold surface, where there is greater availability of nutrients, rather than staying within the scaffolds. This might lead to decreased cell differentiation and myotube formation within the scaffolds instead of the surfaces, as observed in previous studies [26]. To mitigate this issue, scaffolds with optimum nutrient and oxygen diffusibility should be engineered so that the cells stay within the bioprinted scaffold and differentiate. The aim should be to have uniform cell differentiation within and on the scaffold surface. Thus, the degradation and swelling properties of the alginate–gelatin scaffold are critical in terms of promoting overall cell growth kinetics, spreading, and differentiation [26]. 

Interestingly, efforts have been made to increase the degradation kinetics of alginate–gelatin bioinks, even though gelatin is well-known for promoting the degradation kinetics of the scaffolds. Yao et al. [56] incorporated alginate lyase into alginate–gelatin bioinks and enhanced the degradation rate, which resulted in pores that helped in more space for cell adhesion, migration, and cell-cell interaction. On the other hand, the addition of secondary particles can decrease the scaffold’s degradation kinetics and help sustain the alginate–gelatin scaffolds in a liquid medium for a prolonged time. For instance, adding bioglass nanoparticles to alginate–gelatin bioinks can help crosslink more and slow gelatin degradation [54]. Silicate nanoparticles and sodium ions can also stabilize the gelatin molecular structure [57]. However, the amount of particle addition to the hydrogel should be optimized as excess addition could lead to premature or excess gelation of the bioink and high viscosity, affecting the printability of the scaffolds [58]. 

### 3.8. Biocompatibility of the Scaffolds

**Figure 10:**
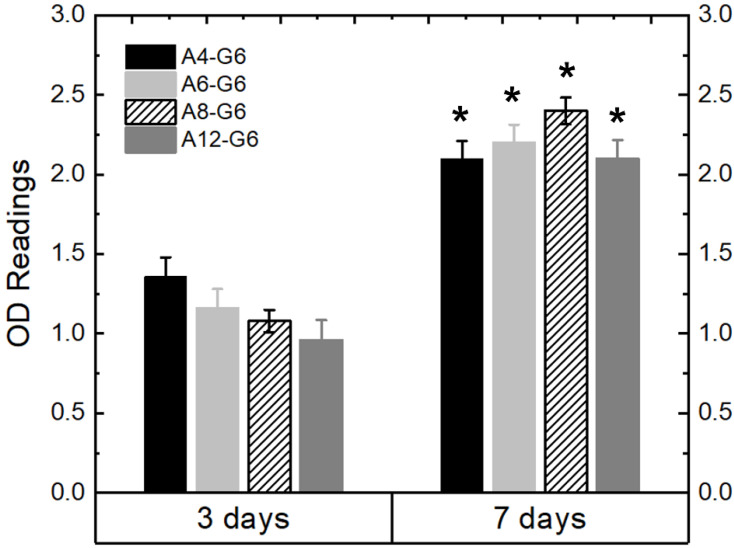
MTT assay results of the various printed scaffolds using C2C12 muscle cells. * means that the OD readings are statistically significant (*p* < 0.05) with respect to the OD readings of the ‘3 days’ specimens.

Figure 10 shows the MTT assay results, indicating the growth kinetics of the C2C12 cells cultured on the various specimens over time. The OD readings indicate a significant (*p* < 0.05) increase in the cell growth kinetics after the 7-day culture period on all the specimens compared to that after the 3-day culture period. The increase in cell growth kinetics over time indicates that the alginate–gelatin compositions are biocompatible and provide a suitable environment for the cells to proliferate. However, the alginate concentration variation slightly influenced the growth kinetics of the C2C12 cells. For instance, after day 3 of the culture, C2C12 cells proliferated less on scaffolds with higher alginate concentrations, probably because of the low bioactivity of alginate. Nonetheless, after day 7 of the culture, cells proliferated irrespective of the alginate concentration on the scaffolds. Overall, all the alginate–gelatin scaffolds explored were biocompatible and suitable for providing a healthy environment for the cells to thrive. In follow-up studies, we will encapsulate the cells within the bioink and analyze their viability over time.

## 4. Conclusions

We successfully developed various alginate–gelatin hydrogel inks and used them in an extrusion bioprinter to develop alginate–gelatin composite scaffolds. It was found that inks with higher alginate concentrations (12% w/v, A12-G6) exhibited good extrudability and were suitable for developing scaffolds with good structural fidelity and print accuracy. The optimum crosslinking treatment of the alginate–gelatin scaffolds was determined to be immersing the scaffolds in a 500 mM CaCl_2_ concentration for 15 min. Post-crosslinking, the A12-G6 scaffolds exhibited excellent structure resolution and fidelity. Notably, the stiffness of the crosslinked A12-G6 scaffolds was similar to that of skeletal muscle. Furthermore, the A8-G6 and A12-G6 scaffolds exhibited satisfactory swelling, indicating that the scaffolds provided a permeable surface and allowed nutrients and oxygen absorption. Finally, the A12-G6 scaffolds showed a sustained degradation rate over 14 days, suggesting that they could survive in vivo. Overall, the A12-G6 scaffold was identified to be the optimum ink formulation for potentially treating skeletal muscle defects. This study’s primary aim was to optimize the acellular ink formulation, validate its printability, determine the best crosslinking parameters, and optimize the basic material properties of the printed scaffolds. With this range of optimized bioink formulations, in follow-up studies, we will incorporate muscle cells into the ink to create a bioink and develop cell-laden ‘live’ constructs for treating VML.

## Figures and Tables

**Table 1:** Formulations and respective nomenclature for the inks and the corresponding bioprinted constructs.

Alginate Concentration (% *w*/*v*)	Gelatin Concentration (% *w*/*v*)	Hydrogel Ink, Construct or Specimen Name
4	6	A4-G6
6	6	A6-G6
8	6	A8-G6
12	6	A12-G6

**Table 2:** Observations of the quality of the A4-G6 bioprinted scaffold based on different printing parameters. The quality (print resolution) of the constructs were rated on a scale from 1 to 5, with 1 being the lowest resolution and 5 being the highest.

Pressure (kPa)	Speed (mm/s)	Quality (1–5)	Observations
7	30	1	No pores, hydrogel spreading
8	30	1	No pores, alginate spreading
9	15	1.5	Some pores formed but poor resolution
10	15	1.5	Some pores formed but poor resolution
>10	Any Speed	1	Hydrogel spreading

**Table 3:** Observations of the A6-G6 bioprinted scaffold based on different printing parameters. The quality (print resolution) of the constructs were rated on a scale from 1 to 5, with 1 being the lowest resolution and 5 being the highest.

Pressure (kPa)	Speed (mm/s)	Quality (1–5)	Observations
7	30	2	Mostly clogged pores, hydrogel spreading
8	30	2.5	Some proper pores were formed, poor resolution
9	25	2	Poor resolution structure with several clogged pores
10	15	3	Some pores formed and moderate resolution
>10	Any Speed	1	Hydrogel spreading

**Table 4:** Observations of the A8-G6 bioprinted scaffold based on different printing parameters. The quality (print resolution) of the constructs were rated on a scale from 1 to 5, with 1 being the lowest resolution and 5 being the highest.

Pressure (kPa)	Speed (mm/s)	Quality (1–5)	Observations
8	30	1	Almost all the pores were clogged
10	30	2	Poor resolution structure with several clogged pores
15	15	2	Pores are formed, but no good borders; poor resolution
16	15	4	Mostly good pores and well-defined borders; good resolution

**Table 5:** Observations of the A12-G6 bioprinted scaffold based on different printing parameters. The quality (print resolution) of the constructs were rated on a scale from 1 to 5, with 1 being the lowest resolution and 5 being the highest.

Pressure (kPa)	Speed (mm/s)	Quality (1–5)	Observations
20	30	2	Good pores but poor resolution with non-uniform extrusion
23	15	2.5	Good pores but moderate resolution with non-uniform extrusion
27	30	3.5	Mostly good pores but improper border outlines
30	15	4.5	Mostly good pores and well-defined borders; good resolution

**Table 6:** Parameters *m* and *n* from a power-law model fit to the flow curves of various gels, prepared with increasing gelatin loading. The overall model fits the experimental data were significant in each case (*p* < 0.05), with all the model parameters also being significant (*p* < 0.01).

		A4-G6	A6-G6	A8-G6	A12-G6
Power-law model	*m* (Pa s)	0.1402	0.284	0.3966	1.4003
	*N*	0.9718	0.9574	0.9773	0.9372
Herschel–Bulkley model	*τ*_0_ (Pa)	−0.1495	−0.4312	−0.6834	−2.7108
	*k* (Pa s)	0.2106	0.5068	0.8008	3.5867
	*N*	0.8979	0.8561	0.8401	0.7413

## Data Availability

Data will be made available on request.
